# Retrospective evaluation of the impact of non-oncologic chronic drug therapy on the survival in patients with bladder cancer

**DOI:** 10.1007/s11096-021-01343-x

**Published:** 2021-11-01

**Authors:** Lisa Haimerl, Dorothea Strobach, Hanna Mannell, Christian G. Stief, Alexander Buchner, Alexander Karl, Tobias Grimm

**Affiliations:** 1 Hospital Pharmacy, University Hospital LMU Munich, Marchioninistr.15, 81377 Munich, Germany; 2 Doctoral Programme Clinical Pharmacy, University Hospital LMU Munich, Marchioninistr.15, 81377 Munich, Germany; 3 Department of Urology, University Hospital LMU Munich, Marchioninistr.15, 81377 Munich, Germany; 4 Department of Urology, Hospital Barmherzige Brüder, Romanstr.93, 80639 Munich, Germany; 5 Urology Practice Kaufbeuren, Gutenbergstraße 8, 87600 Kaufbeuren, Germany

**Keywords:** Bladder cancer, Cystectomy, Medication, Survival, Urothelial carcinoma

## Abstract

*Background* Chronic drug therapy may impact recurrence and survival of patients with bladder cancer and thus be of concern regarding drug choice and treatment decisions. Currently, data are conflicting for some drug classes and missing for others. *Objective* To analyze the impact of common non-oncologic chronic drug intake on survival in patients with bladder cancer and radical cystectomy. *Setting.* Patients with bladder cancer and radical cystectomy (2004–2018) at the University Hospital Munich. *Method* Data from an established internal database with patients with bladder cancer and radical cystectomy were included in a retrospective study. Drug therapy at the time of radical cystectomy and survival data were assessed and follow-up performed 3 months after radical cystectomy and yearly until death or present. Impact on survival was analyzed for antihypertensive, antidiabetic, anti-gout, antithrombotic drugs and statins, using the Kaplan–Meier method, log-rank test and Cox-regression models. *Main outcome measure* Recurrence free survival, cancer specific survival and overall survival for users versus non-users of predefined drug classes. *Results* Medication and survival data were available in 972 patients. Median follow-up time was 22 months (IQR 7–61). In the univariate analysis, a significant negative impact among users on recurrence free survival (*n* = 93; *p* = 0.038), cancer specific survival (*n* = 116; *p* < 0.001) and overall survival (*n* = 116; *p* < 0.001) was found for calcium-channel blockers, whereas angiotensin-receptor-blockers negatively influenced overall survival (*n* = 96; *p* = 0.020), but not recurrence free survival (*n* = 73; *p* = 0.696) and cancer specific survival (*n* = 96; *p* = 0.406). No effect of angiotensin-receptor-blockers and calcium-channel blockers was seen in the multivariate analysis. None of the other studied drugs had an impact on survival. *Conclusion* There was no impact on bladder cancer recurrence and survival for any of the analyzed drugs. Considering our results and the controverse findings in the literature, there is currently no evidence to withhold indicated drugs or choose specific drug classes among the evaluated non-oncologic chronic drug therapies. Thus, prospective studies are required for further insight. *Trail registration* This is part of the trial DRKS00017080, registered 11.10.2019.

## Impact on practice

- The survival of bladder cancer patients after radical cystectomy is not altered by the chronic use of antihypertensives, anticoagulants, antidiabetic drugs, statins or allopurinol. However, data are still limited and prospective studies are urgently needed.
- At present, there is no evidence to withhold any indicated chronic drug therapy for patients with bladder cancer and no recommendation for the use of a specific drug class of e.g. antihypertensives or antidiabetics can be made.
- Optimal treatment for the underlying comorbidities is suggested to be the most important goal irrespective of the drug selection and patients should be encouraged to continue their chronic co-medication and prescription of indicated treatment for chronic diseases should be continued.

## Introduction

Urothelial carcinoma of the urinary bladder (UCB) is the 11^th^ most common cancer worldwide and is characterized by high mortality and recurrence rate [1]. Radical cystectomy (RC) represents the gold standard of intervention for localized muscle-invasive (MIBC) and recurring high-risk non-muscle-invasive bladder cancer (NMIBC) [1]. When first diagnosed, most patients are 70 years or older at which age more than 90% of the patients regularly take drugs [2]. The most often diagnosed comorbidities in cancer patients, e.g. hypertension or diabetes, demand chronic drug therapy [3]. A possible impact of chronic drugs on the risk of UCB development has been discussed for several substances, a prominent example being the thiazolidinedione pioglitazone [4]. While some studies are available on the impact of drugs on the risk of UCB development [5], data regarding an impact on recurrence and survival are still limited. For instance, conflicting results have been found for first-line antihypertensives like angiotensin-converting-enzyme inhibitors (ACEI), angiotensin-receptor-blockers (ARB) and calcium-channel blockers (CCB) [6–12]. Although the precise mechanism remains unclear, the renin–angiotensin–aldosterone system potentially affects carcinogenesis and tumor progression [13]. Of interest, a positive impact of the oral antidiabetic drug metformin has been described in a meta-analysis, but contradicting results have been reported by more recent studies [14, 15]. There are no or very limited data on the impact of other chronically administered drugs, such as additional oral antidiabetics, anticoagulants or anti-gout drugs. On the other hand, in vitro data or biological modelling systems concerning other cancer entities point towards a possible effect of some drug classes on UCB recurrence and progression [16–18]. Low dose ASA is frequently used for the prevention of thromboembolic events in elderly patients. Antineoplastic effects have been discussed for several cancer entities, e.g. colorectal, breast and gastric cancer [19]. Vitamin-K-antagonists are widely used for prophylaxis and therapy for thromboembolic events e.g. in patients with atrial fibrillation or deep vein thrombosis. While studies have found a lower risk for overall cancer incidence in chronic warfarin users [17], no protective effect but a non-significant risk elevation by 27% was described for warfarin on UCB development [20]. Whereas the anti-gout drug allopurinol has been linked to an increased overall cancer incidence, its impact on UCB development remains obscure [21].

While a positive impact of chronically administered non-oncologic drug classes might allow to tailor drug therapy according to patients´ risks, e.g. in the choice of antihypertensive agents, a negative impact could present a therapeutic dilemma when drug therapy of comorbidities is necessary. Thus, more evidence is needed regarding the impact of chronic drug intake on UCB recurrence and survival.

### Aim of the study

To analyze the impact of drug therapy with antihypertensives, antidiabetics, antithrombotics, statins and the anti-gout drug allopurinol on recurrence rate and survival in a retrospective cohort of UCB patients undergoing radical cystectomy.

### Ethics approval

The study was conducted in accordance with the Declaration of Helsinki. Approval was obtained by the ethics committee of the University Hospital Munich (18–427).

## Method

A retrospective observational study was performed at a tertiary teaching hospital using data from an established database of UCB patients at the department of urology of the University Hospital LMU Munich [22]. The database includes UCB patients, who underwent RC between 2004 and 2018.

### Data collection

The following data was collected from the UCB patient database: age, sex, pathological staging (TNM), grading (G), surgical margin (R) and follow-up data on oncologic outcome. The first follow-up was performed three months after RC and continued once yearly with validated questionnaires sent by regular mail, as described previously [22]. Patients were followed-up until present date or until death and recurrence free survival (RFS), cancer specific survival (CSS) and overall survival (OS) were documented [22]. Cause of death was defined by the treating physician or a death certificate. Bladder specimens after RC were examined according to standard histopathological procedures by urogenital pathologists at the Department of Pathology. Tumor staging and grading was undertaken according to the TNM classification and World Health Organization grading criteria [1]. Data on drug therapy from the day of RC were taken from patients` drug charts and added to the database. A pharmacist checked the medication data for completeness and plausibility using the hospitals electronic patient information system (SAP-i.s.h.med, Cerner Corporation, North Kansas City, USA). No additional data on continued or additional drug intake were assessed at follow-ups.

### Analyzed drugs and drug classes

Previously studied non-oncologic drugs and drug classes used for chronic therapy were selected based on their potential effect on UCB development, recurrence or known impact on other cancer entities [6, 9, 10, 12, 15, 17, 23–25]. The following drug classes defined by anatomical therapeutical chemical code (ATC) were included: antihypertensives (ACEI (C09AA), ARB (C09CA), beta blockers (C07AB), CCB (C08)), drugs targeting blood coagulation (acetylsalicylic acid (ASA) (B01AC), vitamin-K-antagonists (B01AA), direct acting oral anticoagulants (DOAC) (B01AF)), antidiabetic drugs (metformin (A10BA02), sulfonylureas (A10BB), thiazolidinediones (A10BG), dipeptidylpeptidase 4 (DPP4)-Inhibitors (A10BH), insulin (A10A)) and miscellaneous (statins (C10AA), allopurinol (M04AA01)). Based on the medication review, patients were classified as user or non-user for every drug class included.

### Statistical analysis

Primary endpoints were RFS and CSS, secondary endpoint was OS. Survival curves were generated by the Kaplan–Meier method and compared by log-rank tests. Hazard ratios (HR) were estimated by Cox analysis with a 95% confidence interval. An univariate analysis was performed for the association between survival and drug intake (user/non-user). If univariate analysis showed a significant result for a drug class, a subsequent multivariate analysis (Cox regression) including other factors predicting survival (age, sex, tumor staging and grading) was performed. A two-sided *p-*value ≤ 0.05 was considered statistically significant. Continuous data are presented as median and interquartile range (IQR). Statistical analyses were performed and figures created with Microsoft Excel®2016 (Seattle, WA, USA) and MedCalc®17 software (MedCalc, Ostend, Belgium).

## Results

### Patient characteristics

Overall, for 972 patients with UCB undergoing RC, follow-up and medication data were available and OS and CSS were analyzed. In addition, for 859 patients, data on recurrence were available and RFS analysis was performed. The median follow-up time was 22 months (IQR 7–61 months, max. 170 months). Patient characteristics are presented in Table 1.

**Table 1:** Patient characteristics

Variables	*n* = 972
Age [years], median (IQR)	70 (62–76)
*Sex, n (%)*	
Female	230 (24)
Male	742 (76)
*Tumor stage and grade, n (%)*	
pT0	84 (9)
pTa	23 (2)
pTis	123 (13)
pT1	75 (8)
pT2	201 (21)
pT3	320 (33)
pT4	130 (13)
pTX	16 (2)
pN0	613 (63)
pN +	240 (25)
pNX	119 (12)
M0	886 (91)
M1	86 (9)
G1-2 / low grade	81 (8)
G3 / high grade	792 (82)
Grade unknown	99 (10)
*Surgical margin, n (%)*	
Negative (R0)	835 (86)
Positive (R1)	130 (13)
Unknown (RX)	7 (1)
*ASA-Status, n (%)*	
1	26 (3)
2	367 (38)
3	559 (58)
4	14 (1)
Unknown	6 (1)
*Smoking history, n (%)*	
Yes	474 (49)
No	404 (42)
Unknown	94 (10)

*T*: tumorstage; *G*: grade; *N*: lymph nodes; *M*: metastasis; *ASA-Status*: American Society of Anesthesiologists physical status classification system

### Influence of age, sex, tumor staging and grading on survival of patients with UCB

As seen in Table 3, T-stage, N-status and presence of metastasis (TNM), R-status and age each had an independent influence on RFS, CSS and OS in the multivariate analysis. No independent impact on RFS, CSS and OS was found for tumor grade (G) and sex.

### Influence of antihypertensive drugs on recurrence and survival of patients with UCB

**Fig. 1:**
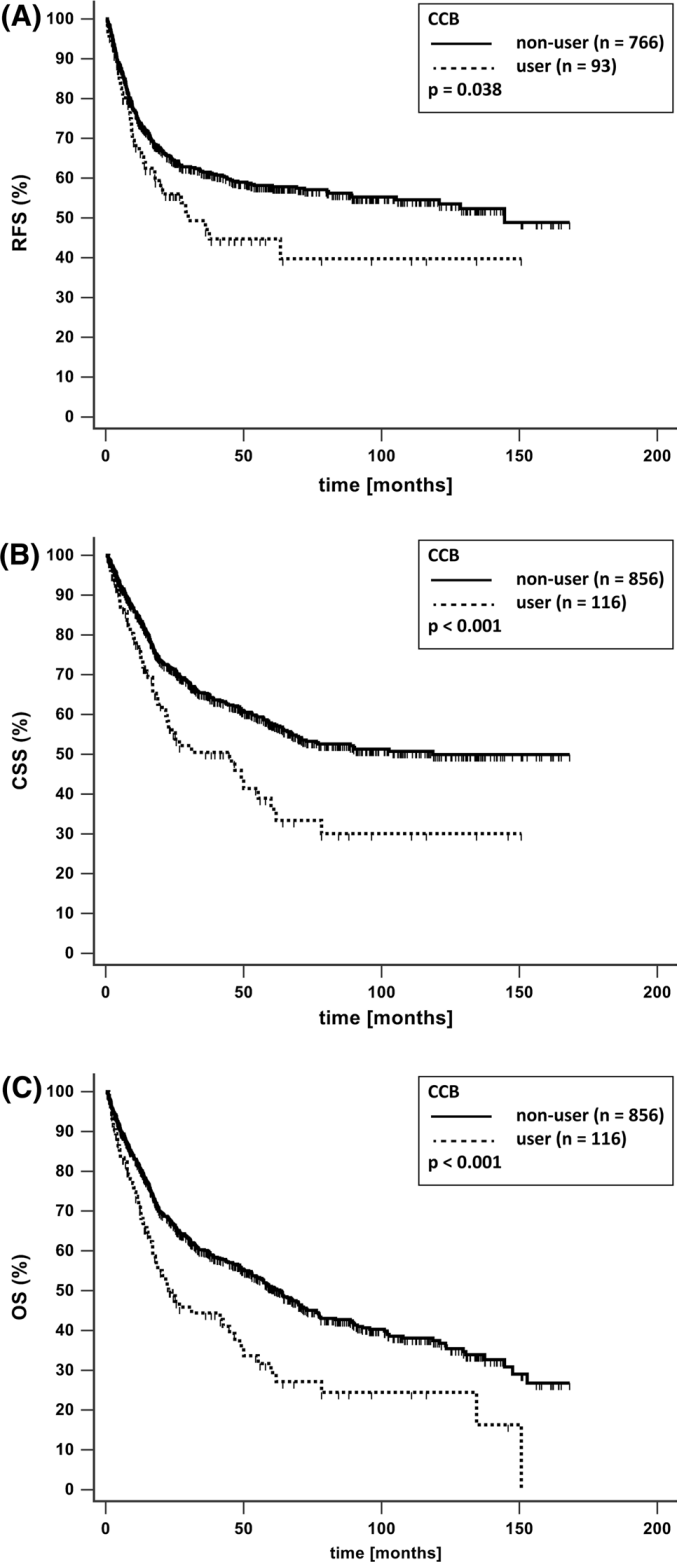
Kaplan–Meier curves with and without calcium-channel blocker (CCB) use for **a** recurrence free survival (RFS) **b** cancer specific survival (CSS) and **c** overall survival (OS)

**Fig. 2:**
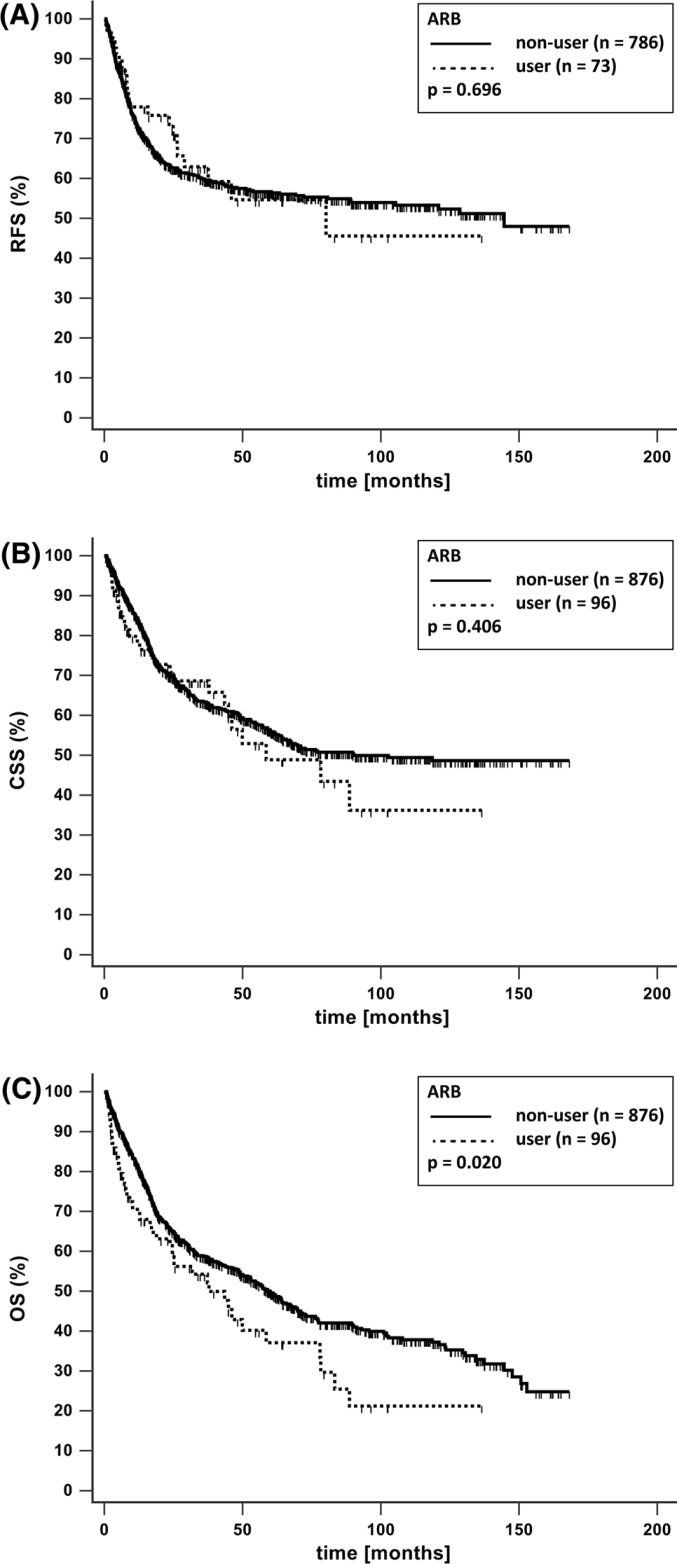
Kaplan–Meier curves with and without angiotensin receptor blocker (ARB) use for **a** recurrence free survival (RFS) **b** cancer specific survival (CSS) and **c** overall survival (OS)

Regarding antihypertensives, 286 patients took beta blockers, 274 ACEI, 96 ARB and 116 patients CCB (Table 2). Comparing users with non-users in an univariate analysis, no impact on RFS, CSS and OS was found for ACEI and beta blockers (Table 2). Of note, CCB had a statistically significant negative impact on RFS, CSS and OS (Fig. 1). The median survival of non-users versus users was 145 compared to 30 months for RFS, 119 versus 45 months for CSS and 63 versus 24 months for OS. ARB had a significant negative impact on OS, but no impact on RFS (Fig. 2). The median OS of non-users versus users was 59 compared to 38 months. To test whether the drugs, which were shown to influence survival in patients with UCB in the univariate analysis, have an impact on RFS, CCS and OS in combination with other factors predicting survival in these patients, we performed a multivariate Cox regression analysis. ARB and CCB, which had a negative impact on several parameters in the univariate analysis, did not show any independent impact on RFS, CSS or OS in the multivariate analysis (Table 3). Antihypertensives without significant result in the univariate analysis were not considered in the multivariate analysis.

**Table 2:** Univariate analysis comparing recurrence free survival (RFS), cancer specific survival (CSS) and overall survival (OS) between users and non-users of the listed drugs

	RFS			CSS			OS		
	User (*n*)	Non-user (*n*)	*p* value	User (*n*)	Non-user (*n*)	*p* value	User (*n*)	Non-user (*n*)	*p* value
*Antihypertensives*									
ACE-Inhibitors	216	643	0.921	274	698	0.829	274	698	0.479
Angiotensin receptor blockers	73	786	0.696	96	876	0.406	96	876	0.020*
Beta blockers	238	621	0.720	286	686	0.821	286	686	0.313
Calcium-channel blockers	93	766	0.038*	116	856	< 0.001*	116	856	< 0.001*
*Antidiabetic Drugs*									
Insulin	21	838	0.651	25	947	0.578	25	947	0.226
Metformin	45	814	0.457	57	915	0.507	57	915	0.526
Sulfonylureas	16	843	0.091	21	951	0.171	21	951	0.155
DPP4-Inhibitors	14	845	0.908	16	956	0.811	16	956	0.797
Thiazolidinedione	3	856	–^a^	6	966	–^a^	6	966	–^a^
*Anticoagulants*									
Acetylsalicylic acid	198	661	0.771	246	726	0.107	246	726	0.069
Vitamin-K-antagonists	30	829	0.209	36	936	0.175	36	936	0.855
Direct acting oral anticoagulants	18	841	0.409	23	949	0.219	23	949	0.894
*Miscellaneous*									
Allopurinol	54	805	0.959	67	905	0.845	67	905	0.246
Statins	174	685	0.653	203	769	0.296	203	769	0.482

*p ≤ 0.05; ^a^No statistical analysis performed due to insufficient patient numbers

**Table 3:** Multivariate analysis: Cox regression model predicting (a) recurrence free survival (RFS) (b) cancer specific survival (CSS) and (c) overall survival (OS)

Variables	*p* value	HR	95% CI
*(a) Recurrence free survival (n = 614)*			
T-stage (pT34 vs. pT < 3)	< 0.001*	2.29	1.70–3.09
N-status pN + vs. pN0	< 0.001*	1.69	1.26–2.26
Metastases (M1 vs. M0)	< 0.001*	3.06	2.15–4.36
Grade (G3 / high grade vs. G1-2 / low grade)	0.702	0.92	0.59–1.42
R-status (R1 vs R0)	< 0.001*	1.84	1.30–2.59
Age (cont.)	0.002*	1.02	1.01–1.04
Sex (male vs. female)	0.934	1.01	0.74–1.39
Calcium-channel blockers (user vs. non-user)	0.946	1.01	0.69–1.49
*(b) Cancer specific survival (n = 744)*			
T-stage (pT34 vs. pT < 3)	< 0.001*	2.35	1.77–3.13
N-status pN + vs. pN0	< 0.001*	1.82	1.39–2.37
Metastases (M1 vs. M0)	< 0.001*	2.35	1.68–3.27
Grade (G3 / high grade vs. G1-2 / low grade)	0.820	1.05	0.69–1.61
R-status (R1 vs R0)	< 0.001*	2.02	1.49–2.75
Age (cont.)	< 0.001*	1.03	1.01–1.04
Sex (male vs. female)	0.834	0.97	0.73–1.30
Calcium-channel blockers (user vs. non-user)	0.146	1.30	0.91–1.85
*(c) Overall survival (n = 744)*			
T-stage (pT34 vs. pT < 3)	< 0.001*	2.19	1.71–2.80
N-status pN + vs. pN0	< 0.001*	1.80	1.42–2.27
Metastases (M1 vs. M0)	< 0.001*	2.20	1.63–2.97
Grade (G3 / high grade vs. G1-2 / low grade)	0.650	1.09	0.75–1.58
R-status (R1 vs R0)	< 0.001*	1.87	1.41–2.47
Age (cont.)	< 0.001*	1.03	1.02–1.04
Sex (male vs. female)	0.998	1.00	0.77–1.30
Calcium-channel blockers (user vs. non-user)	0.136	1.27	0.93–1.75
Angiotensin receptor blockers (user vs. non-user)	0.117	1.33	0.93–1.90

*HR*: hazard ratio; *CI*: confidence interval

*p ≤ 0.05

### Influence of antidiabetic drugs on recurrence and survival of patients with UCB

In our cohort, 57 patients were treated with the oral antidiabetic metformin, 25 with insulin, 21 with sulfonylureas, 16 with DDP4-inhibitors and 6 with thiazolidinediones (Table 2). No association between the intake of insulin, metformin, sulfonylureas and DDP4-inhibitors and survival (RFS, CCS and OS) was found (Table 2) in comparison to non-users in the univariate analysis. The small number of patients treated with thiazolidinediones (*n* = 6) precluded a statistical analysis for this drug class. Since antidiabetics showed no effect in the univariate analysis, they were not included in the multivariate analysis.

### Influence of other frequently used drugs on recurrence and survival of patients with UCB

No association between the anticoagulants ASA, vitamin-K-antagonists and DOACs and RFS, CCS and OS was found (Table 2). Additionally, for the cholesterol lowering statins as well as for the anti-gout drug allopurinol, no correlation to RFS, CCS or OS was found. With no significant impact in univariate analysis, these drugs were not included in multivariate analysis.

## Discussion

We retrospectively analyzed the impact of various common non-oncologic drugs on RFS, CSS and OS in UCB patients undergoing RC. In the multivariate analysis, no effect was found for intake of several classes of first-line antihypertensives, statins, the anti-gout drug allopurinol and antithrombotic drugs.

ACEI and ARB are first-line drugs for the treatment of hypertension according to national and international guidelines [26, 27]. Previous retrospective studies have shown improved outcomes for patients with NMIBC and MIBC taking ACEI or ARB. However, studies were small with 40 to 143 patients [6–8, 11]. In our study, we were able to analyze a higher number of patients with 274 on ACEI and 96 on ARB. In addition, the aforementioned studies mostly did not analyze ACEI and ARB separately, thus possibly masking differences in their impact on cancer recurrence and survival. Some evidence exists that this might be of importance, although the mechanism is unknown. A study evaluating the risk for urinary tract cancer development enrolling over 30,000 patients found no effect for ACEI, while ARB increased the risk [10]. Recently, a Finnish retrospective study evaluating the impact of antihypertensives on survival after bladder cancer diagnosis found no effect for ACEI, but an improved survival for ARB [9]. Of note, the positive ARB effect was only significant in the multivariate analysis for men, but not women. Our results agree with the Finnish findings concerning ACEI, but not for ARB (Table 2). Unfortunately, we were unable to differentiate the effect of ARB intake by sex, since only 16 of the 73 patients were female (data not shown). In contrast to our study focusing on patients with RC, the Finnish evaluation included all UCB patients, independent of the chosen treatment.

While beta blockers represented the most common antihypertensives in our analysis, no impact on RFS, CSS or OS was found. This is in line with findings by Dal Moro et al. (20 patients) and Santala et al. (500 patients) [8, 9].

For the antihypertensive drug class CCB, less data on the risk for UCB progression is available so far. In our study, use of CCB did have a negative impact on RFS, CSS and OS in the univariate analysis, while no effect was seen in the multivariate analysis. In the study by Dal Moro et al. only 14 patients received CCB and there was no significant effect on UCB recurrence [8].

Taken together, there are still conflicting results concerning a possible impact of different classes of antihypertensives on recurrence and survival in UCB patients but in our opinion, these effects may be neglected. For UCB development, hypertension has been identified as a risk factor (32% risk increase) with the effect being statistically significant in women and non-significant in men [28]. Dal Moro et al. found arterial hypertension to be a risk factor for UCB recurrence [8]. Thus, it has been discussed that appropriate treatment of hypertension is important, regardless of the antihypertensive drug class [9]. As a limitation of our study we were unable to include clinical outcome of the antihypertensive treatment in our analysis due to the retrospective design.

Low dose ASA was associated with an improved 5-year CSS and OS in a study including 1061 UCB patients with RC. In a multivariate analysis, ASA use was associated with lower cancer specific mortality and all-cause mortality [25]. In our study, including 246 ASA users, we could not confirm these findings. However, a possible trend towards an improved outcome for OS could be seen (*p* = 0.069, Table 2). Interestingly, Pastore et al. found a decrease in UCB recurrence after transurethral resection of the bladder for patients taking low dose ASA for at least two years, although the effect was no longer present with additional statin therapy [12]. Thus, additional diseases and co-medications may alter or mask an effect of low-dose ASA on UCB recurrence.

Of note, our study is the first analyzing the impact of vitamin-K-antagonist use on recurrence and outcome of UCB. Among the 36 patients taking phenprocoumon, we did not find an impact on UCB outcome.

To our knowledge, there are no studies available concerning a potential impact of DOAC on UCB outcome. No impact on recurrence and survival was found for the 23 patients in our cohort. Nevertheless, we expect that a rising number of UCB patients will take DOAC, as the risk for venous thrombosis after RC is high and DOAC are increasingly used for prophylaxis of cancer associated thrombosis [29]. Thus, more data on this issue can be expected in the near future.

In our study, insulin and oral antidiabetic drugs, except thiazolidinediones, did not show any impact on recurrence or survival in UCB patients. Pioglitazone, the only currently available thiazolidinedione in Germany, has been broadly discussed for an increased risk of bladder cancer development [4]. The facts are less clear for other oral antidiabetics. Diabetes without intake of the biguanide metformin has been described as a risk factor for recurrence and survival in patients with UCB [30]. A meta-analysis summarizing nine retrospective studies found a positive impact of metformin on RFS and CSS, but not OS [14]. Currently, a phase-II-study is investigating oral metformin in patients with NMIBC [31]. In contrast, a more recent population-based cohort study found no impact of metformin intake on CSS in UCB patients in a multivariate analysis [15]. In addition, the authors describe a negative impact for the sulfonylurea glyburide and no effect for other oral antidiabetics and insulin [15]. However, a lack of effect on UCB recurrence and survival for oral antidiabetic drugs and insulin was also described by others [32]. We assume that, like in hypertension, the adequate therapy of diabetes is the goal, which might reduce recurrence and improve survival in UCB patients.

Concerning allopurinol, a study from Taiwan found an increased overall cancer incidence for users, but no effect on UCB development [18]. To our knowledge, our study is the first analyzing the survival of UCB patients using allopurinol. No effect was found on recurrence and outcome in our cohort of 67 allopurinol users.

In coherence with a previous study [33], no effect on UCB recurrence and survival was seen for statins in our cohort. Conflicting results have been reported by others [12, 34, 35]. A direct and immediate anticancer effect of statins, described for several cancer entities in observational studies, has recently been questioned and explained as merely based on selection and immortal-time bias [36].

Considering the results of our study and the, in many aspects controversial, findings of other evaluations, a possible impact of chronic non-oncologic drug therapy on recurrence and survival in UCB patients seems unlikely. Even for drugs or drug classes with the most published data like metformin and antihypertensives, the evidence is conflicting. Several reasons might account for this. First, differences in the analyzed patient cohorts, time of observation and country-specific therapy guidelines may play a role. Second, the small number of patients included in some analyses might lead to statistical correct, but clinical misguiding results. In addition, as seen for some antihypertensive drug classes, gender effects may play a role, which have not been studied yet. Most important, all studies reporting on drug effects on UCB recurrence and survival so far, including the study presented here, are of a retrospective design. Finally, the duration and dosage of drug intake was not considered in our study, as is the case with most studies. Thus, prospective studies with adequate cohort sizes, following patients for an appropriate time and collecting data regarding drug exposure (duration and dosage) are urgently needed to gain more reliable data on the impact of chronic drugs on UCB recurrence and survival.

## Conclusion

Considering the results of our and previous studies, there is currently no reason to withhold an indicated chronic drug therapy with antihypertensives, anticoagulants, statins, allopurinol or antidiabetics in patients with UCB and RC. However, data on many drugs and drug classes are still limited and the available evidence is based on small numbers and retrospective data. Despite these limitations, adequate treatment of concomitant diseases like hypertension and diabetes mellitus might be an important point for survival in UCB patients. Given the evidence available so far, no recommendation for a specific drug class of antihypertensives or antidiabetics is possible today.
